# Electrokinetic Proton Transport in Triple (H^+^/O^2−^/e^−^) Conducting Oxides as a Key Descriptor for Highly Efficient Protonic Ceramic Fuel Cells

**DOI:** 10.1002/advs.202004099

**Published:** 2021-03-25

**Authors:** Arim Seong, Junyoung Kim, Donghwi Jeong, Sivaprakash Sengodan, Meilin Liu, Sihyuk Choi, Guntae Kim

**Affiliations:** ^1^ School of Energy and Chemical Engineering Ulsan National Institute of Science and Technology (UNIST) Ulsan 44919 Republic of Korea; ^2^ Department of Chemistry University of Liverpool Liverpool L69 7ZD UK; ^3^ Department of Materials Imperial College London London SW7 2BX UK; ^4^ School of Materials Science and Engineering Georgia Institute of Technology Atlanta GA 30332‐0245 USA; ^5^ Department of Aeronautics Mechanical and Electronic Convergence Engineering Kumoh National Institute of Technology Gyeongbuk 39177 Republic of Korea

**Keywords:** perovskite, proton electrokinetics, proton tracer diffusion coefficient (D^*^_H_), protonic ceramic fuel cells, triple conducting oxides

## Abstract

Recently, triple (H^+^/O^2−^/e^−^) conducting oxides (TCOs) have shown tremendous potential to improve the performance of various types of energy conversion and storage applications. The systematic understanding of the TCO is limited by the difficulty of properly identifying the proton movement in the TCO. Herein, the isotope exchange diffusion profile (IEDP) method is employed via time‐of‐flight secondary ion mass spectrometry to evaluate kinetic properties of proton in the layered perovskite‐type TCOs, PrBa_0.5_Sr_0.5_Co_1.5_Fe_0.5_O_5+_ *_*δ*_* (PBSCF).Within the strategy, the PBSCF shows two orders of magnitude higher proton tracer diffusion coefficient (*D* ^*^_H_, 1.04 × 10^−6^ cm^2^ s^−1^ at 550 °C) than its oxygen tracer diffusion coefficient at even higher temperature range (*D* ^*^_O,_ 1.9 × 10^−8^ cm^2^ s^−1^ at 590 °C). Also, the surface exchange coefficient of a proton (k*_H_) is successfully obtained in the value of 2.60 × 10^−7^ cm s^−1^ at 550 °C. In this research, an innovative way is provided to quantify the proton kinetic properties (*D* ^*^_H_ and k*_H_) of TCOs being a crucial indicator for characterizing the electrochemical behavior of proton and the mechanism of electrode reactions.

## Graphical abstract

Triple (H^+^/O^2−^/e^−^) conducting oxides (TCOs) have shown tremendous potential to improve the performance of various energy conversion and storage applications. However, the direct visualization of proton movement has not been investigated. Herein, the isotope exchange diffusion profile method is employed to evaluate proton kinetic properties (*D* ^*^_H_ and *k* ^*^_H_) in TCOs being crucial indicator for highly efficient protonic ceramic fuel cells.

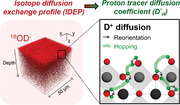

Ceramic fuel cells, an electrochemical device for the direct conversion of chemical fuel to electricity, have obtained much attention with several advantages such as high energy conversion efficiencies, low pollutant emissions, and excellent fuel flexibility.^[^ ^1^ ^]^ Among them, protonic ceramic fuel cell (PCFC) using proton conducting oxide (PCO) as an electrolyte is considered as a promising candidate to operate in low‐to‐intermediate temperature range (400–600 °C) because of the higher ionic conductivity of PCO compared to that of pure oxygen conducting electrolytes, such as yttria stabilized zirconia, Sr‐and Mg‐doped lanthanum gallate, and gadolinium doped ceria.^[^ ^2^ ^]^ Moreover, the water formation reaction occurs at the cathode by the movement of protons from fuel anode to air cathode, achieving high fuel efficiency without fuel dilution. Despite these attractive advantages of PCFC, its practical use and commercialization are still limited due to the large cathodic polarization resistance.

Generally, the cathodic resistance of PCFC is determined by the combined reaction rate with oxygen electroreduction and water formation, written globally as Equation (1)

(1) $$\begin{array}{cc} & \;\frac{1}{2}O_{2}\left(\mathrm{gas}\right)+2e^{-}\left(\mathrm{cathode}\right)\\ & \;\;+2H^{+}\left(\mathrm{electrolyte}\right)\;\to \;H_{2}O\left(\mathrm{gas}\right) \end{array}$$

Accordingly, in order to reduce the large cathodic polarization resistance, the cathode properties such as fast oxygen electroreduction rates and simultaneously high electrochemical activities for H^+^, O^2−^, and e^−^ must be secured.^[^ ^3^ ^]^ Such materials have been called triple‐conducting oxides (TCOs) which can make the entire sites of bulk cathode a reactive site, resulting in higher performance. Thus, recent studies have been focused on the development of novel TCO materials. For example, various transition metals (e.g., Co and Fe) were doped into the pure proton conductive oxide (BaZr_1−_ *_x_*Y*_x_*O_3_) at high concentration level to improve electronic conductivity and oxygen ion conduction such as BaCo_0.4_Fe_0.4_Zr_0.1_Y_0.1_O_3−_ *_*δ*_*.^[^ ^4^ ^]^ A promising candidate of the TCO is cation‐ordered double perovskite materials (AA'B_2_O_5+_ *_*δ*_*, A = Ln, A’ = Ba and B = transition metals) with their high triple conductivities (H^+^, O^2−^, and e^−^) and high hydration properties (e.g., PrBa_0.5_Sr_0.5_Co_2−_ *_x_*Fe*_x_*O_5+_ *_*δ*_*).^[^ ^5^ ^]^

Although good TCO cathode materials have been reported, the fundamental behavior of proton in TCOs is not yet fully understood because of the following challenges. First, due to the high electronic transference number (approaching unity) of some TCOs, it is very difficult to separate proton conductivity from the total electrical conductivities including contributions of O^2−^, H^+^, and e^−^ using a conventional measurement based on concentration cells (i.e., the electromotive force method).^[^ ^6^ ^]^ Second, under typical PCFC operating conditions, the interactions between H^+^, O^2−^, and e^−^ make it difficult to analyze the kinetic properties of protons in TCOs. Recently, several studies have been reported the proton chemical diffusion coefficient of TCOs by the electrical conductivity relaxation in water partial pressure gradient.^[^ ^7^ ^]^ However, this technique includes the effect of movement of different diffusion species (e.g., O^2−^, and e^−^) due to the consumption of oxygen vacancies, redistributing all the carrier related to conductivity. Thus, some more advanced technique is required to directly visualize protonics in TCOs without other diffusion species.

In this regard, we employ the isotope exchange diffusion profile (IEDP) method in which deuterium was used as a tracking indicator of proton diffusion via time‐of‐flight secondary ion mass spectrometry (ToF‐SIMS).^[^ ^8^ ^]^ This strategy enables to precisely evaluate the proton tracer diffusion coefficient (D^*^_H_) analysis of triple conducting oxide, PrBa_0.5_Sr_0.5_Co_1.5_Fe_0.5_O_5+_ *_*δ*_* (PBSCF), excluding the interactions of oxide ions and electrons. The proton diffusion coefficient value of PBSCF at 550 °C (*D* ^*^_H_, 1.04 × 10^−6^ cm^2^ s^−1^) is two orders of magnitude higher than its oxygen diffusion coefficient at 590 °C (*D* ^*^_O_, 1.9 × 10^−8^ cm^2^ s^−1^). The *D* ^*^_H_ of PBSCF shows higher value than *D* ^*^_O_ of typical mixed ionic and electronic conductors (MIECs) such as Ba_0.5_Sr_0.5_Co_0.8_Fe_0.2_O_3−_ *_*δ*_* (BSCF), PrBaCo_2_O_5+_ *_*δ*_* (PBCO), GdBaCo_2_O_5+_ *_*δ*_* (GBCO), La_0.6_Sr_0.4_Co_0.2_Fe_0.8_O_3−_ *_*δ*_* (LSCF), and La_0.6_Sr_0.4_CoO_3−_ *_*δ*_* (LSC) (1.63 × 10^−9^–3.01 × 10^−11^ cm^2^ s^−1^) at 550 °C. Furthermore, we have successfully obtained the surface exchange coefficient of a proton (*k* ^*^_H_) in the value of 2.60 × 10^−7^ cm s^−1^. Based on the outstanding proton kinetics of PBSCF, the excellent electrochemical performance is obtained in practical PCFC condition (e.g., 0.42 W cm^−2^ at 500 °C). In this study, we introduce an innovative way to quantify proton kinetic properties (*D* ^*^_H_ and *k* ^*^_H_) of TCOs being significant indicator for characterizing the electrochemical behavior of proton and the mechanism of electrode reactions.

MIEC based layered perovskite, PBSCF, was selected as a model system to study the proton kinetic properties in TCOs because of its high electrical conductivity and outstanding hydration properties.^[^ ^5^, ^9^ ^]^ To determine the surface exchange coefficient and bulk proton diffusivity of the PBSCF sample, isotope‐exchange treatment was performed under precise control of temperature and deuterium oxide (D_2_O) vapor pressure. **Figure** **1** is a schematic illustration, explaining how deuterium defects form and diffusion occurs in the perovskite‐type structure (a–d). The deuterium conduction is based on the existence of deuterium defects at a moderate temperature and dissociative absorption of D_2_O, which requires the presence of oxygen vacancies (Figure 1a). As a next step, D_2_O dissociates into D^+^ and OD^−^ and the OD^−^ ion fills the oxygen vacancy (Figure 1b). Then, the deuterium forms a covalent bond with the lattice oxygen and thus, the proton defects are formed (Figure 1c). This process can be written according to the reaction below^[^ ^10^ ^]^

(2) $$D_{2}O+V_{O}^{\cdot \cdot }+O_{O}^{x}↔2{\mathrm{OD}}_{O}^{\cdot }$$

**Figure 1:**
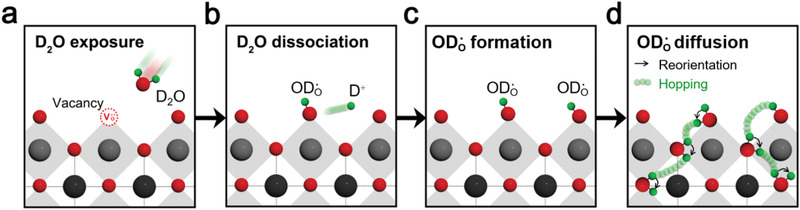
Schematic illustration of proton defect formation and diffusion in D_2_O exchange condition in perovskite‐type structure. Red circles = oxide ions (O^2−^), black circles = A‐site cations, gray circles = B‐site cations, and green circles = deuteriums (D^+^). a) Exposure of perovskite‐type oxide in D_2_O‐containing air. b) Dissociation of D_2_O into D^+^ and ${\mathrm{OD}}_{O}^{\cdot }$. c) Proton defect formation. d) Deuterium diffusion by reorientation and hopping.

After 2${\mathrm{OD}}_{O}^{\cdot }$ defect formation, the deuterium diffusion (Figure 1d) proceeds in two basic steps. The first step is a reorientation step, bending the 2${\mathrm{OD}}_{O}^{\cdot }$ group toward the adjacent oxygen. Next step, in deuterium transfer, deuterium migrates by hopping between neighboring lattice oxygens ($O_{O}^{\times }$) through the destruction and formation of hydrogen bonds. In this regard, we intentionally introduce the deuterium defects as a tracking indicator of proton diffusion, providing a quantitative analysis of proton kinetics. To illustrate our strategy for the investigation of proton kinetic properties of PBSCF, the entire process from the preparation of the sample to ToF‐SIMS analysis is outlined in **Figure** **2a**. The dense PBSCF pellet (typical density ≈98.5% of theoretical) was polished for uniform D_2_O adsorption onto the surface, followed by heat‐treatment under 10 vol% D_2_O‐containing air at 250–550 °C. Surface mass spectrum analysis and IEDP measurements were performed on the D_2_O‐exchanged PBSCF sample.

**Figure 2:**
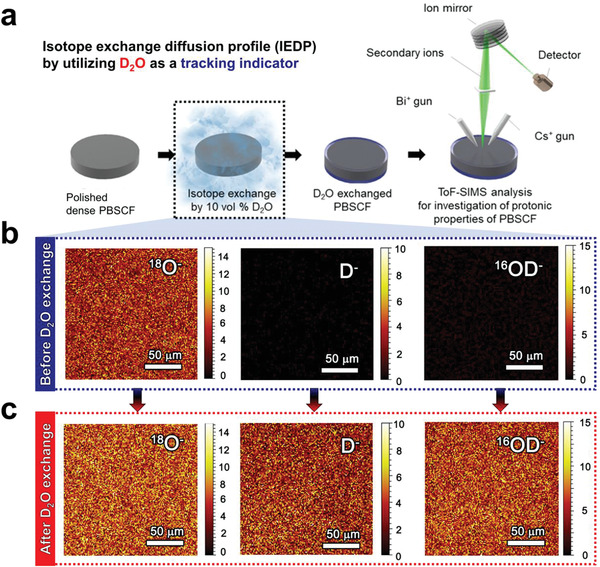
a) Schematic illustration of preparation and incorporation of D_2_O for the ToF‐SIMS measurement. Secondary ion mapping images of ^18^O^−^, D^−^, and ^16^OD^−^ for the surface of PBSCF pellet b) before and c) after D_2_O exchange.

Before the ToF‐SIMS measurement, X‐ray diffraction (XRD) patterns were collected to identify the chemical stability of PBSCF crystalline structure in D_2_O exchange condition. As shown in Figure S1 in the Supporting Information, the PBSCF sample exhibits a layered perovskite structure without any secondary phase. After exposure to a 10 vol% D_2_O containing air at 600 °C for 24 h, the XRD pattern of the PBSCF was examined with no structural change, showing excellent chemical stability of PBSCF. In Figure S2 in the Supporting Information, atomic force microscopy revealed that the dense PBSCF surface has an root mean square (rms) roughness (R_q_) of 22.7 nm, supporting density and surface quality of samples. Additionally, after ToF‐SIM analysis, we measured the crater depth of the PBSCF pellet at ≈351 nm to identify the depth of profiling using a surface profilometer (Figure S3, Supporting Information).

Figure S4 in the Supporting Information presents a normalized ToF‐SIMS spectrum of the PBSCF sample before and after the D_2_O‐exchange. As clearly shown in the ToF‐SIMS mass spectrum, the D^−^ (*m z* ^−1^ = 2.014) and ^16^OD^−^ (*m z* ^−1^ = 18.009) signals are successfully detected from the surface of D_2_O‐exchanged PBSCF, and the mass resolution is sufficient to separate between the ^16^OD^−^ and ^18^O^−^ (in nature, *m z* ^−1^ = 17.999). Therefore, D^−^ and ^16^OD^−^ peaks are observed after the D_2_O‐exchange treatment, indicating that the exchange process successfully forms D^−^ and ^16^OD^−^ ions on the surface. The incorporation of protons on the surface of the PBSCF sample is also visualized by secondary ion mapping (Figure 2b,c), where bright yellow and dark red colors represent the high and low concentration of observed ion species, respectively. While the ^18^O^−^ species are similarly observed before and after the D_2_O‐exchange, the D^−^ and ^16^OD^−^ species are clearly observed over the entire surface of the PBSCF sample only after D_2_O exchange, indicating that the proton defect formation reaction (Equation (2)) homogeneously occurs on the surface.

As shown in **Figure** **3a**, the 3D‐mapping image of OD^−^ ion intensity can be visualized in the 50 × 50 µm of the analysis area for the D_2_O‐exchanged PBSCF sample (annealed at 350 °C). Figure 3b,c shows the normalized OD^−^ tracer diffusion profiles and these experimental data can be fitted to Fick's second law with the semi‐infinite model since the in‐diffusion of deuteron was shorter than half of the PBSCF sample thickness

(3) $$\begin{array}{cc} C^{\prime }\left(x\right)=\; & \mathrm{erfc}\left(\frac{x}{2\sqrt{D^{*}t}}\right)-\;\exp\left(hx+h^{2}D^{*}t\right)\\ & \times \mathrm{erf}\left\{\left(\frac{x}{2\sqrt{D^{*}t}}\right)+\left(h\sqrt{D^{*}t}\right)\right\} \end{array}$$

where *h* is the ratio of the surface exchange coefficient (*k* ^*^) to the diffusion coefficient (*D* ^*^) and *t* is the anneal time. The *C’*(*x*) is the normalized deuteron isotopic concentration calculated from

(4) $$C^{\prime }\left(x\right)=\;\frac{C\left(x\right)-C_{\mathrm{bg}}}{C_{g}+C_{\mathrm{bg}}}$$

where *C(x)*, *C*_g_, and *C*_bg_ are deuteron fraction, deuteron concentration in air, and background isotope fraction, respectively. The calculated *D* ^*^ and *k* ^*^ values are presented in Figure 3d,e, and Table S1 in the Supporting Information. The diffusion coefficient of the proton (*D* ^*^_H_) increases with increasing temperature due to the thermally activated motion of the proton (Figure 3d). Interestingly, unlike the surface exchange behavior of O^2−^ for general MIEC materials, the surface exchange coefficient of the proton (*k* ^*^_H_) is reduced with increasing temperature (Figure 3e). This behavior can be explained by the exothermic properties of proton uptake at the surface, validating our approach for proton kinetics.^[^ ^11^ ^]^ The proton diffusion coefficient (*D* ^*^_H_) of PBSCF shows excellent value of 1.04 × 10^−6^ cm^2^ s^−1^ at 550 °C which is two orders of magnitude higher than its oxygen diffusion coefficient at 590 °C (*D* ^*^_O_, 1.9 × 10^−8^ cm^2^ s^−1^).^[^ ^11^ ^]^ Also, *D* ^*^_H_ value of PBSCF is much higher than the O^2−^ diffusion coefficients (D^*^_O_) of other representative SOFC cathode materials (Figure 3f), such as BSCF, PBCO, GBCO, LSCF, and LSC (1.63 × 10^−9^–3.01 × 10^−11^ cm^2^ s^−1^) at 550 °C.^[^ ^8^, ^13^ ^]^

**Figure 3:**
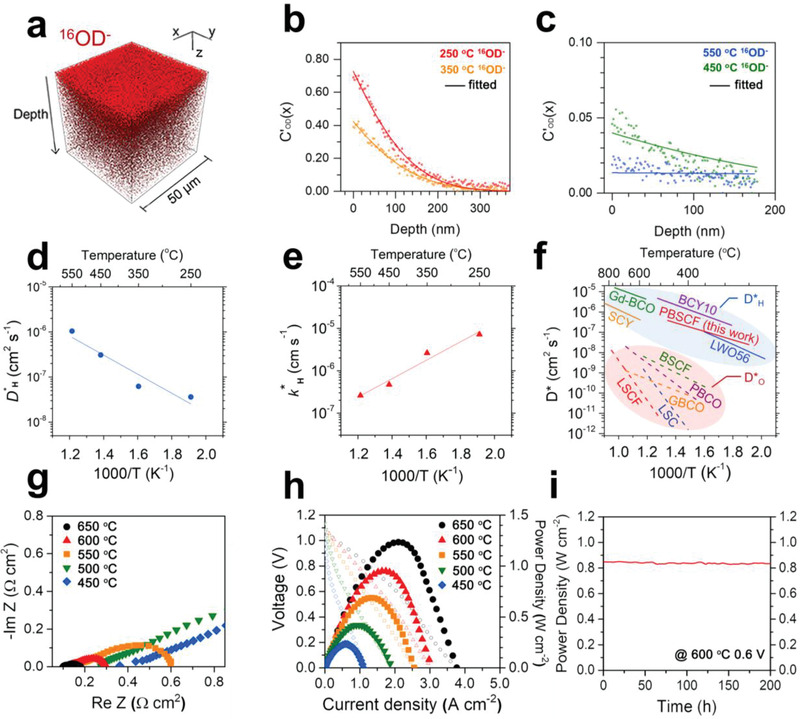
a) 3D‐mapping image of ^16^OD^−^ ion intensity in the 50 × 50 µm^2^ of the analysis area for D_2_O‐exchanged PBSCF sample annealed at 350 °C. b,c) Normalized ^16^OD^−^ depth profiles obtained from D_2_O‐exchanged PBSCF annealed at a temperature range of 250–550 °C. The solid lines represent the best fit to the Fick's second law. d,e) Diffusion and surface kinetics coefficient of proton (*D* ^*^_H_ and *k* ^*^_H_) values of the PBSCF sample. f) Comparison of diffusion coefficients (*D* ^*^_H_) of the PBSCF with other representative MIEC materials: Ba_0.5_Sr_0.5_Co_0.8_Fe_0.2_O_3−_ *_*δ*_* (BSCF),^[^ ^12^ ^]^ PrBaCo_2_O_5+_ *_*δ*_* (PBCO),^[^ ^8^ ^]^ GdBaCo_2_O_5+_ *_*δ*_* (GBCO),^[^ ^13^ ^a]^ La_0.6_Sr_0.4_Co_0.2_Fe_0.8_O_3−_ *_*δ*_* (LSCF),^[^ ^13^ ^d]^ La_0.6_Sr_0.4_CoO_3−_ *_*δ*_* (LSC),^[^ ^13^ ^b]^ La_27.15_W_4.85_O_55.28_V_0.73_ (LWO),^[^ ^13^ ^c]^ BaCe_0.9_Y_0.1_O_3−_ *_*δ*_* (BCY10),^[^ ^13^ ^e]^ (Ba_0.965_Gd_0.035_)(Ce_0.935_Gd_0.035_)O_3−_ *_*δ*_* (Gd‐BCO),^[^ ^13^ ^f]^ and SrCe_0.95_Yb_0.05_O_3−_ *_*δ*_* (SCY).^[^ ^13g^ ^]^ Lines in the red and blue circle represent *D* ^*^ values for oxygen ion and proton, respectively. g) Impedance spectra of the single cell (PBSCF/BZCYYb/NiO‐BZCYYb) measured under open‐circuit condition. h) *I*–*V* curve and corresponding power density curves of the single cell. i) Long‐term stability data of the single cell applying a constant voltage of 0.6 V at 600 °C.

In PCFC system, the proton migration (bulk and/or surface) would be more significant indicator rather than hydration property in the cathodic reaction due to the sufficiently introduced protons from the electrolyte. Consequently, fast proton migration rates of cathode could result in the high electrochemical performances of PCFC. In this respect, we prepared anode‐supported single cells with a configuration of PBSCF/BZCYYb/NiO‐BZCYYb to investigate the effect of outstanding proton kinetic properties of PBSCF cathode. The chemical compatibility between PBSCF and BZCYYb was investigated by XRD pattern of pellet heat‐treated at 950 °C for 4 h by screen‐printing PBSCF slurry onto BZCYYb/NiO‐BZCYYb bilayer. No secondary peaks occur, indicating the absence of a reaction between two phases (Figure S5, Supporting Information). As shown in Figure S6 in the Supporting Information, the BZCYYb electrolyte was 15.7 µm thick and dense without any detectable pinhole. Also, the porous cathode microstructure was 14.8 µm thick and the interfaces between the electrodes and the electrolyte seems to be well connected. The A.C. impedance spectra and current–voltage (*I*–*V*) curves of single cell were obtained at various temperatures (Figure 3g,h). Generally, impedance spectra of a single cell are used to describe all resistances associated with the electrolyte and electrode. The intercept with real axis at high frequency is the ohmic resistance originated from electrolyte, whereas the difference between the low‐ and high‐frequency intercepts represents the electrode polarization resistance. As can be expected from the favorable protonic kinetic properties of the PBSCF in our experimental results, the polarization resistances are 0.080, 0.166, and 0.437 Ω cm^2^ at 650, 600, and 550 °C, respectively. Based on low polarization resistance, maximum power density of 1.23, 0.95, and 0.69 W cm^−2^ were obtained at 650, 600, and 550 °C, respectively. Notably, the PBSCF single cell shows a power density of 0.42 W cm^−2^ even at 500 °C, which is much higher than the required power density of 0.25 W cm^−2^ for efficient operation.^[^ ^14^ ^]^ At a constant voltage of 0.6 V, as shown in Figure 3i, the PBSCF/BZCYYb/NiO‐BZCYYb single cell shows stable power output with 0.85 W cm^−2^ for a break‐in period of 200 h.

In the present study, we first employed an innovative way to evaluate proton kinetic properties of layered perovskite type TCOs, PBSCF, via ToF‐SIMS. Using deuterium as a tracking indicator, proton formation and transport were observed on both surface and bulk of the PBSCF. The IEDP of deuterium is successfully applied to reveal the proton diffusion coefficient (*D*_H_^*^, 1.04 × 10^−6^ cm^2^ s^−1^ at 550 °C) and the surface exchange coefficient of the proton (*k* ^*^_H_, 2.60 × 10^−7^ cm s^−1^ at 550 °C) of PBSCF. Benefiting from the fast proton transfer ability, the PBSCF cathode shows excellent electrochemical performance for PCFC operation at low temperatures (e.g., 0.42 W cm^−2^ at 500 °C). Quantitative characterization of the proton kinetics in TCO can be an important indicator providing a scientific basis for the rational design of highly efficient electrode materials of PCFCs.

## Conflict of Interest

The authors declare no conflict of interest.

## Supporting information

Supporting Information
