# Supplementary material for: Electrokinetic Proton Transport in Triple (H+/O2−/e−) Conducting Oxides as a Key Descriptor for Highly Efficient Protonic Ceramic Fuel Cells
Source: Adv Sci (Weinh). 2021 Mar 25;8(11):2004099. doi: 10.1002/advs.202004099 (PMC8188232; doi:10.1002/advs.202004099)
Supplement: Supplementary file 1 — Supporting Information [file ADVS-8-2004099-s001.pdf]

© 2021 Wiley-VCH GmbH

ADVANCED SCIENCE
Open Access

Supporting Information

for *Adv. Sci.*, DOI: 10.1002/advs.202004099

Electrokinetic proton transport in triple ($H^+/O^{2-}/e^-$) conducting oxides as a key descriptor for highly efficient protonic ceramic fuel cells

Arim Seong, Junyoung Kim, Donghwi Jeong, Sivaprakash Sengodan, Meilin Liu,
Sihyuk Choi*, and Guntae Kim*

Supporting information

Electrokinetic proton transport in triple ($H^+/O^{2-}/e^-$) conducting oxides as a key descriptor for highly efficient protonic ceramic fuel cells

Arim Seong[+], Junyoung Kim[+], Donghwi Jeong, Sivaprakash Sengodan, Meilin Liu, Sihyuk Choi, and Guntae Kim**

A. Seong, Dr. J. Kim, D. Jeong, Prof. G. Kim
School of Energy and Chemical Engineering
Ulsan National Institute of Science and Technology (UNIST)
Ulsan, 44919, Republic of Korea
E-mail: gtkim@unist.ac.kr

Dr. J. Kim
Department of Chemistry
University of Liverpool
Liverpool, L69 7ZD, UK

Prof. S. Sengodan
Department of Materials
Imperial College London
London, SW7 2BX, UK

Prof. M. Liu
School of Materials Science & Engineering
Georgia Institute of Technology
Atlanta, GA 30332-0245, USA

Prof. S. Choi
Department of Mechanical Engineering
Kumoh National Institute of Technology

Gyeongbuk 39177, Republic of Korea
E-mail: sh.choi@kumoh.ac.kr

Prof. S. Choi
Department of Aeronautics, Mechanical and Electronic Convergence Engineering
Kumoh National Institute of Technology

Gyeongbuk 39177, Republic of Korea
E-mail: sh.choi@kumoh.ac.kr

+ Arim Seong and Junyoung Kim contributed equally to this work

1. Experimental Section

1.1. Synthesis of samples

The Pechini method was used to synthesize $PrBa_{0.5}Sr_{0.5}Co_{1.5}Fe_{0.5}O_{5+\delta}$ (PBSCF). The desired composition was obtained by each dissolving nitrate salts in distilled water with the addition of ethylene glycol and quantitative amounts of citric acid. After a viscous resin was formed, the mixture was heated to 280 ℃ in the air followed by combustion to make fine powders, which were pre-calcined for 4 hours at 600 ℃ and ball-milled for 24 h in acetone. The typical solid-state reaction (SSR) was used to synthesize $BaZr_{0.1}Ce_{0.7}Y_{0.1}Yb_{0.1}O_{3-\delta}$ (BZCYYb) powders. Stoichiometric amounts of barium carbonate, cerium oxide, zirconium oxide, yttrium oxide powders, and ytterbium oxide (all from Aldrich Chemicals) were mixed by ball milling process for 24 h with yttria-stabilized zirconia balls using ethanol. After drying the ethanol at 80 ^{o}C, the powder was calcined at 1100 ℃ for 10 h in the air (10 ^{o}C min^{-1} for heating and cooling rate). The milling and calcination steps were repeated one more cycle to confirm phase formation. The glycine nitrate process (GNP) was used for synthesizing the NiO for the anode. Stoichiometric amounts of nitrates with a proper amount of glycine were dissolved in distilled water. The solutions were heated up to 350 ℃ and followed by combustion to make fine powders.

1.2. Preparation of samples for structural analysis

The phases of synthesized materials were investigated by an X-ray diffractometer (Rigaku diffractometer, Cu Kα radiation). The pre-calcinated powder of PBSCF and BZCYYb were sintered at 1150 ^{o}C 4 h and 1600 ^{o}C 4 h, respectively. To investigate the chemical stability of PBSCF in humidified conditions, the PBSCF phase was checked after steam exposure (1 0 vol% H_2O containing air) for 24 h at 600 ℃. To examine the chemical reactivity b

etween the PBSCF and BZCYYb, PBSCF slurry was screen-printed onto BZCYYb pe llet, followed by sintered at 950 ^{o}C 4h. The microstructures and cross-section images of the PBSCF/BZCYYb/NiO-BZCYYb single cell was observed using field emission s canning electron microscopy (Nova Nano SEM, FEI, USA).

1.3. Fabrication of electrochemical single cell

The anode-supported cell (PBSCF/BZCYYb/NiO-BZCYYb) was fabricated for the measurement of the electrochemical performance. The calcined PBSCF was blended with a binder (Heraeus V006) for air electrode slurries. The NiO-BZCYYb anode was prepared by a mixture of NiO and BZCYYb (weight ratio of 6.5:3.5) after being ball-milled for 24 h in ethanol. The BZCYYb suspension was applied to the NiO-BZCYYb support by drop-coating, followed by drying in the air and treated by heat at 400 ^{o}C for 1 h to remove organics. As a next step, sintering was followed up in a two-step protocol that the sample was exposed to 1550 ^{o}C for 2 min and then 1400 ℃ for 4 h to maximize the growth of grain while minimizing the Ba evaporation. PBSCF cathode slurry was screen-printed onto the surface of the BZCYYb electrolyte and was finally sintered 950 ℃ in the air for 4 h. Ag wires were attached to both electrodes and electrical behavior was measured in a pseudo-four probe configuration

1.4. Isotope exchange and ToF-SIMS measurements

The PBSCF was pressed to pellets with a diameter of 20 mm and thickness in the range 0.5-1 mm. The pellets were sintered at 1150 ^{o}C for 24 h (typical density ~98.5 % of theoretical) and roughness of the sintered pellet was measured by atomic force microscopy (AFM, Multimode V, Veeco). The isotope proton exchange was performed under accurate control of temperature in the range of 250~550 ℃ and of vapor pressure with 10 vol% D_2O-containing air. The bubbler containing D_2O was heated at 47 ^{o}C using a heating tape to obtain 10 vol%

D_2O.The D_2O-exchanged samples were analyzed by time-of-flight secondary ion mass spectrometry (ToF-SIMS) on an Ion ToF-SIMS 5 (ION-TOF GmbH, Münster, Germany). A 25 keV Bi^+ primary ion beam of 1.10 pA current was used to generate the secondary ions for analysis and a Cs^+ beam (2 kV) incident for sputtering. The energetic Cs^+ ions form dipoles on the surface of sample, generating electric field in the surface. Therefore, when the accelerated particles move out, the electrons are going out together into a flight path towards detector. Because of this surface negative ionization phenomena, the investigated all elements have one negative charge (*i.e.*, $^{18}O^-$, D^-, OD^-). Deuterium depth profiles were investigated from the exchanged surface of the sample by sputter depth profiling. After ToF-SIMS analysis, the crater depth was measured using a KLA-Tencor P6 surface profilometer.

1.5. Electrochemical performance test

For the single-cell tests, Ag wires were attached at the cathode and anode side using Ag paste (SPI supplies, Product 05063-AB) as a current collector. The NiO-BZCYYb anode-supported single cell was sealed fully onto one end of the alumina tube using a ceramic adhesive (Aremco, Ceramabond 552). Humidified H_2 (3% H_2O) was applied to the anode side as a fuel through a water bubbler with a flow rate of 100 mL min^{-1}, while the air was supplied as an oxidant to the cathode during the single-cell test. The impedance spectra and *I-V* curves were acquired with a BioLogic Potentiostat and analyzed with EC-lab software at an operating temperature from 450 to 650 ^oC in intervals of 50 ^oC. The impedance spectra were obtained at open-circuit voltage condition. The current stability was measured under a constant voltage of 0.6 V at 600 °C.

2. Supplementary Results

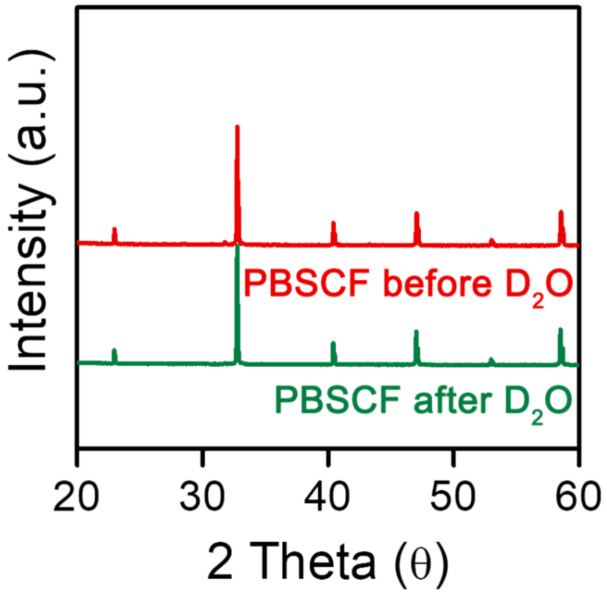

Supplementary Figure 1. X-ray diffraction patterns of PBSCF sintered at 1150 ℃ for 24 h, before and after D_2O exposure (10 vol% D_2O containing air) for 24 h at 600 ℃.

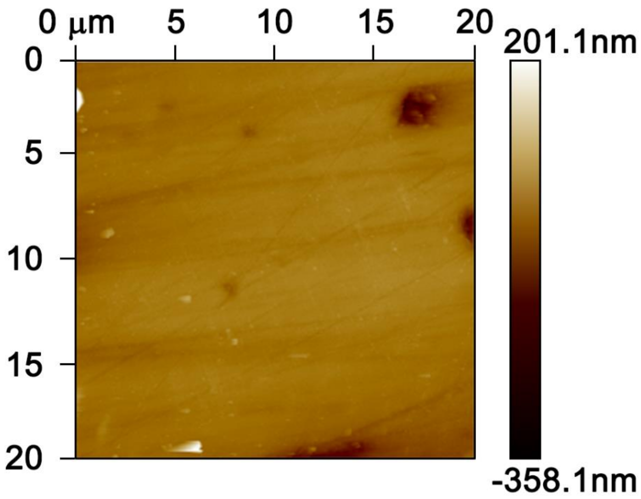

Supplementary Figure 2. Atomic force micrographs of PBSCF sample with a root-mean-squared roughness of 22.7 nm.

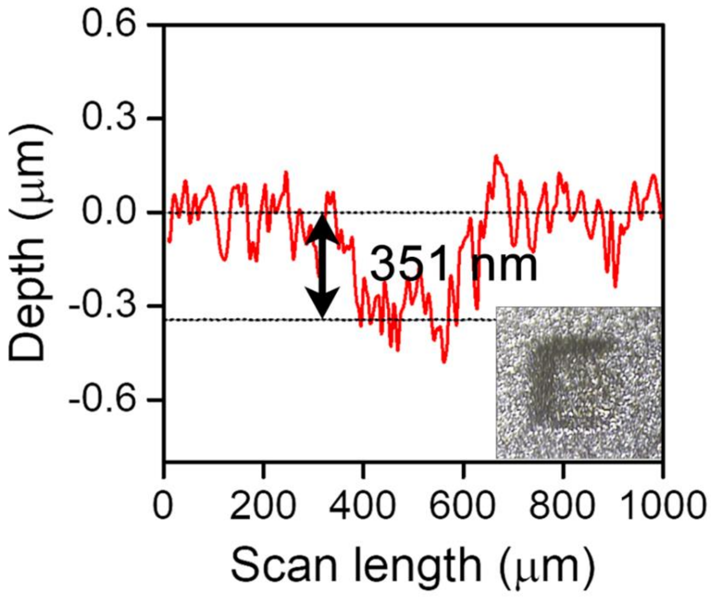

Supplementary Figure 3. Surface profile data of the PBSCF pellet after ToF-SIMS measurement. Inset image shows the crater on the PBSCF surface after Cs^+ beam sputtering.

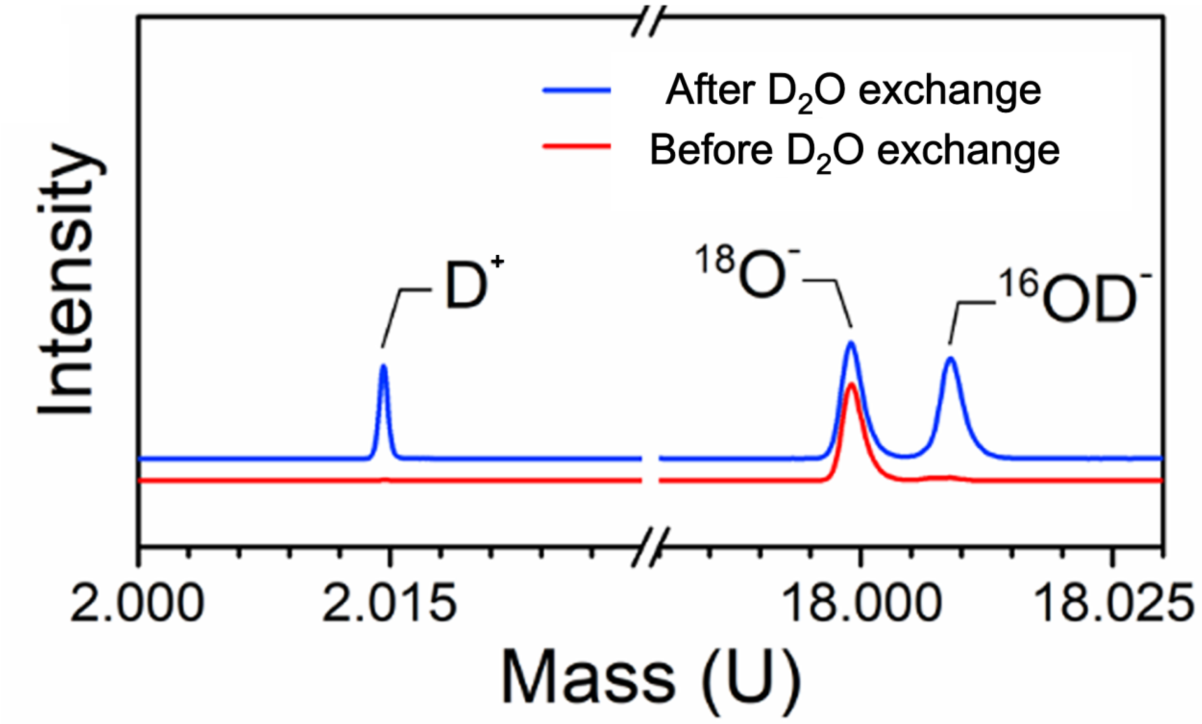

Supplementary Figure 4. Protonic properties of the surface of PBSCF pellet measured by ToF-SIMS. Normalized mass spectrum analysis of a D_2O-exchanged PBSCF around U = 2 and 18.

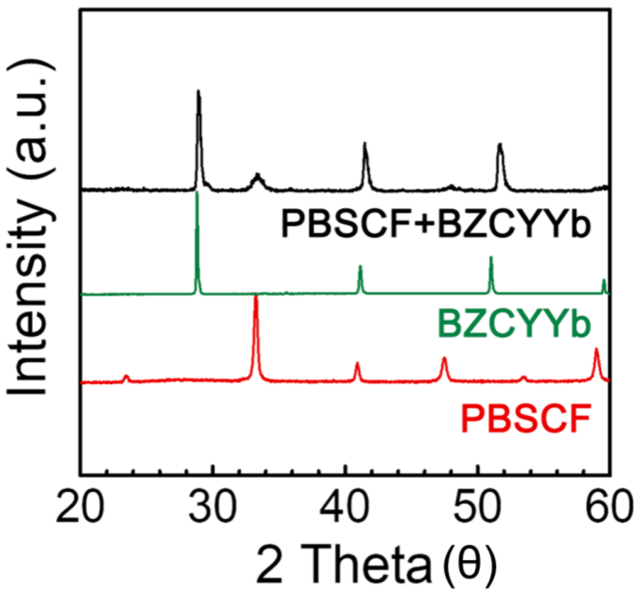

Supplementary Figure 5. XRD differaction pattern of PBSCF sintered at 1150 ℃ for 4 h, BZCYYb sintered at 1600 ℃ for 4 h, and screen-printed PBSCF on BZCYYb/NiO-BZCYYb bi-layer sintered at 950 ℃ for 4h.

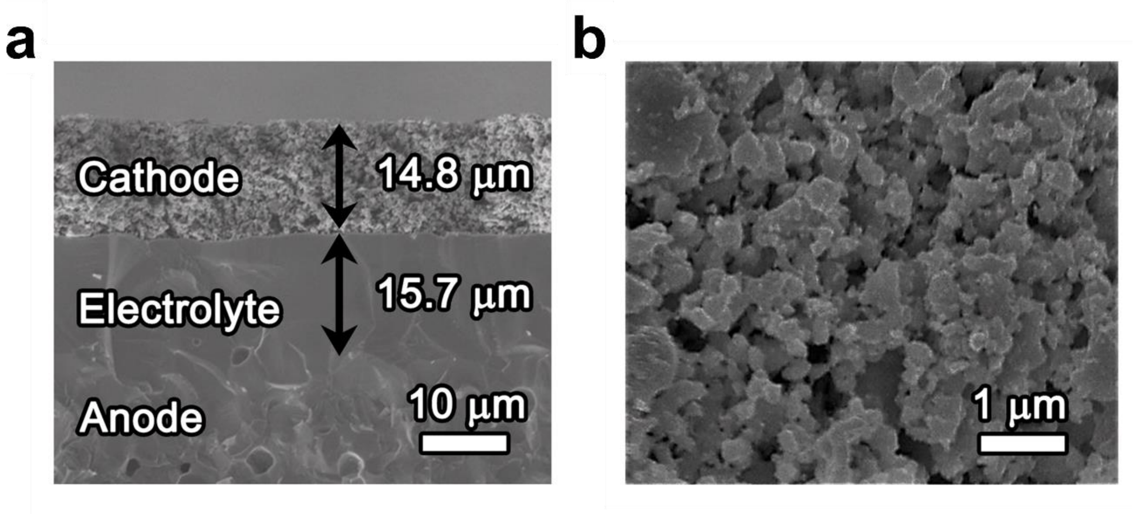

Supplementary Figure 6. a) Cross-sectional SEM image of PBSCF/BZCYYb/NiO-BZCYYb cell. **b)** SEM image of porous PBSCF cathode; For the single cell test, humidified hydrogen (3 vol% H_2O) and air were used as a fuel and oxidant, respectively.

Supplementary Table 1. D^{*}_{H} and k^{*}_{H} values of D_2O-exchanged PBSCF sample annealed at various temperatures

Temperature (℃)	D^{*}_{H} (cm^2 s^{-1})	k^{*}_{H} (cm s^{-1})
250 ℃	3.62×10^{-8}	7.18×10^{-6}
350 ℃	6.23×10^{-8}	2.63×10^{-6}
450 ℃	3.10×10^{-7}	4.70×10^{-7}
550 ℃	1.04×10^{-6}	2.60×10^{-7}
